# Relocation at older age: results from the Cognitive Function and Ageing Study

**DOI:** 10.1093/pubmed/fdv050

**Published:** 2015-04-28

**Authors:** Yu-Tzu Wu, A. Matthew Prina, Linda E. Barnes, Fiona E. Matthews, Carol Brayne

**Affiliations:** 1 Department of Public Health and Primary Care, Institute of Public Health, Forvie Site, University of Cambridge, School of Clinical Medicine, Cambridge Biomedical Campus, Cambridge CB2 0SR, UK; 2 Centre for Global Mental Health, Institute of Psychiatry, King's College London, De Crespigny Park, Denmark Hill, London SE5 8AF, UK; 3 MRC Biostatistics Unit, Institute of Public Health, University of Cambridge, Cambridge CB2 0SR, UK

**Keywords:** environment, communities, epidemiology, relocation, older age

## Abstract

**Background:**

Community environment might play an important role in supporting ageing in place. This paper aims to explore relocation at older age and its associations with individual and community level factors.

**Methods:**

The postcodes of the 2424 people in the year-10 interview of the Cognitive Function and Ageing Study (CFAS) in England were mapped onto Enumeration Districts and linked to their corresponding Townsend deprivation score and the 2011 rural/urban categories. Multilevel logistic regression was conducted to examine the influence of the baseline individual (age, gender, education and social class) and community (rural/urban categories and area deprivation) level factors on relocation over 10 years.

**Results:**

One-third of people moved residence after the age of 65 years and over. Older age, low education, low social class and living in rural areas at baseline were associated with higher probability of moving later in life. The likelihood of relocation in later life increased from least to most deprived areas (odds ratio: 2.0, 95% confidence interval: 1.4, 2.8).

**Conclusions:**

Urban/rural contexts and area deprivation are associated with relocation at older age and indicate that community environment may be relevant to ageing in place.

## Introduction

Relocation is a stressful life event, and even more so at older age, a period when moving is common.^1^ The percentage of relocation in people aged 50 or above has been reported to be between 30 and 50%.^2–5^ Although some people might change their residence in early older age after retirement (‘first move’), relocation at older age has been related to decline of physical and cognitive functions (‘second or third moves’).^5,6^ Older people may need increased support through living with family, friends or moving to care settings involuntarily. Involuntary relocation at older age can be related to physiological or psychological disturbances and difficulties of regaining attachment and emotional connection to new residences.^7–9^ The concept of ‘ageing in place’, which supports older people remaining living in their local environments could be beneficial to healthy ageing and has become a key research interest and policy area of the UK government and other developed countries.^10,11^

The Environmental Press Theory has suggested that people with reduced individual competence are more sensitive to stress from environments which may lead to maladaptive behaviours.^12^ Most existing studies in gerontology have focused on improving housing environment or care homes for ageing populations, but few have examined the potential influence of community environment, composed of ‘pull and push’ factors, on relocation in later life.^13,14^ Environmental press in urban communities, such as noise, heavy traffic, high level of crime and deprivation, might ‘push’ older people to move away from their urban residences. Based on the UK Census 2011, a higher proportion of people aged 65 or above lived in rural areas compared with the general population.^15^ However, lack of local services is a known barrier for older people to remain living in their communities independently.^4,15,16^ The characteristics of community environment might influence the probability of ageing in place.^17^ Using a longitudinal population-based study with a large sample size can provide the opportunity to explore the pattern of relocation over 10 years and the potential impact of community level factors.

### Aims of this study

The study aims to describe the pattern of relocation in older age and explore whether certain types of community environments increase the probability of relocation causing potential barriers to ageing in place. Specific objectives included:

- to investigate the proportion of people relocating at older age and the direction of the relocation (from rural to urban areas or from urban to rural areas);
- to explore the association between relocation and individual (socio-demographic factors) and community (rural/urban categories, deprivation score) level factors;
- to examine potential modified effects of urban/rural contexts on the association between relocation and area deprivation.

## Methods

### Study population

This study was based on the Medical Research Council Cognitive Function and Ageing Study (MRC CFAS), a longitudinal population-based study investigating cognitive and physical decline of people aged 65 and over in six centres across England and Wales (Cambridgeshire, Gwynedd, Newcastle upon Tyne, Nottingham, Oxford and Liverpool).^18^ One centre with non-identical methods is excluded from this analysis (Liverpool). Community and institutionalized populations were sampled from General Practice Registers with an equal size of age group 65–74 and 75 years or over. The baseline interview was conducted between 1991 and 1994 and delivered by trained interviewers visiting the participants' residence. Among 16 258 invited for the study, 13 004 people completed the initial screening interview with response rate 80%. The main follow-up waves included 1, 2, 6 and 8-year rescreen or assessment of the partial sample and 10-year follow-up of the total sample. The year-10 follow-up in 2001 included all the survivals from the baseline. The interview was conducted in participants' residences including a detailed assessment of mental status and self-reported information on chronic conditions. Among 6767 eligible participants, 3145 people completed the interview with response rate 72% (2392 died before completing the interview, 951 refused and 279 moved away). Owing to the lack of comparable information on environmental data, this study included the 2424 participants from the four English centres (Cambridgeshire, Newcastle upon Tyne, Nottingham and Oxford), which contain a wide variety of community environments. The participants in Cambridgeshire were more likely to live in more rural settings with sparse density of population and property, while the other three centres were in more urban settings. Nottingham and Newcastle are considered to be more deprived areas than Cambridgeshire and Oxford.

### Enumeration district and relocation in older age

The Enumeration District (ED) is a geographical unit used in the UK census before 2001. Postcodes of the baseline participants were mapped to the 1991 ED code using National Statistics Postcode Directory (NSPD).^19^ The mapping method was applied to the participants of the year-10 interview. Information on relocation over 10 years was based on comparison of old and new ED codes. People with the same ED code in 1991 and 2001 were assumed to be living at the same residence or areas for over 10 years. Different ED codes indicate relocation after 65 years of age. A small proportion (1.9%) of people with missing ED codes at the baseline had unknown status of relocation and were excluded from the analysis.

### Individual level measurements

Socio-demographic information, including age, gender, education and social class, were recorded during the baseline interview and included in the analyses to explore whether the likelihood of relocation was different across these subgroups. Education was divided into two groups: people with 9 or fewer years of education, and those with 10 years or more. The longest occupation reported by the respondents was used to classify social class of each participant according to Registrar General's occupation-based social class.^20^ The participants with social class classification I to IIINM were considered as non-manual, while social class IIIM to V were categorized as manual. Being male, high education and non-manual social class were treated as reference groups.

### Community level measurements

Baseline Townsend deprivation score on ED level was calculated in a previous study.^19^ The deprivation score aggregates four household/individual socioeconomic indicators, including the proportion of unemployed individuals aged 16–64, the proportion of households without a car, with more than one person per room and that are not owner-occupied. Higher score indicates more deprived areas.^21^ The least deprived quartile was used as the reference. To obtain comprehensive information of urban and rural areas, the ED codes in 1991 and 2001 were linked to the 2011 Rural/Urban Classification for Small Area Geographies using the NSPD information. The 2011 version provides more detailed classification including three urban categories (Major Conurbation, Minor Conurbation, City and Town) and two rural categories (Town and Fringe, Villages and Dispersed).^22^ A sensitivity analysis was conducted to investigate whether using the 2011 version compared with the 2001 version had any substantial influence on the findings.

### Analysis strategy

This study first investigated the distributions of the five rural/urban categories in 1991 and 2001 to provide an overview of community environments over these 10 years. The rural/urban categories in people with different ED codes were compared to explore the direction (from urban to rural/from rural to urban) of relocation in older age. Multilevel logistic regression was used to calculate odds of relocation in older age according to different individual (age, gender, education and social class) and community level (rural/urban categories and area deprivation) factors. Interaction terms of urban/rural areas and deprivation index were added to the regression model to examine the modified effect of urban/rural contexts on the associations between area deprivation and relocation in older age.

## Results

### The proportion and direction of relocation in older age

The distributions of the five rural/urban categories were similar at the baseline and 10-year follow-up. Nearly, 40% of the respondents lived in City and Town areas, 40% in Major and Minor Conurbation areas and 20% in the two rural categories (Town and Fringe, Village and Dispersed).

**Fig. 1:**
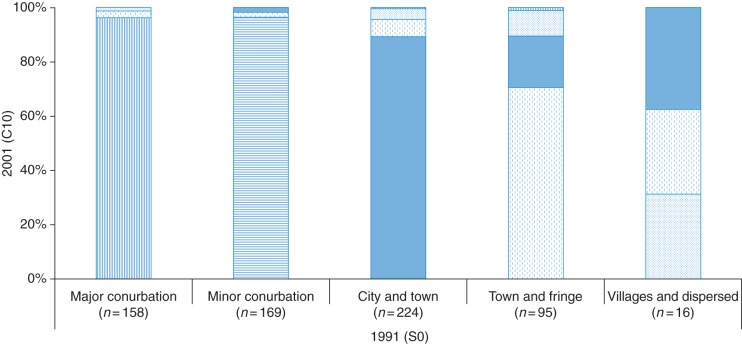
The direction of relocation: people relocating between the baseline and year 10 and their rural/urban categories (*n* = 662, 27.8%). Each pattern represents one rural/urban category. Straight line indicates major conurbation, horizontal line indicates minor conurbation, plain represents city and town, dash denotes town and fringe and dot indicates villages and dispersed. The proportion of people moved to different (various pattern) categories or within same (same pattern) rural/urban categories over 10 years; for example, in the Town and Fringe 1991, over 70% of people moved within the same category (dash); nearly 19% of people living in Town and Fringe in 1991 moved to City and Town (plain) in 2001.

Among the 2424 participants, 1716 (70.8%) individuals had the same ED code at baseline and year-10 follow-up and 662 (27.3%) people had different ED code from 1991. There were 46 (1.9%) people with missing ED codes at the baseline. The direction of relocation was reported in Fig. 1. The majority of relocated people (*n* =662) moved within the same category. About 20% of people living in rural areas (Town and Fringe, Villages and Dispersed) in 1991 moved to the three urban categories (Major Conurbation, Minor Conurbation, City and Town) in 2001. This is a small number of individuals (*n* =25). Nearly, 6% (*n* = 32) of people living in urban areas at baseline moved to rural areas during these 10 years.

### Factors impacting on relocation in older age

A significantly higher proportion of relocation was found in the older age group, those with less education and lower social class (Table 1). Of those who responded to the year-10 interview, the percentage of relocation gradually increased from younger (24.6%) to older (36.7%) age groups.

**Table 1:** Socio-demographic and environmental factors for individuals who had or had not moved over 10 years (*n* = 2424)

	*Relocation, *n* (%)*		**P*-value of *χ*^2^ test*
	*No*	*Yes*	
Overall (missing=46)	1716 (72.2)	662 (27.8)	
Age^a^			
74–79	735 (75.4)	240 (24.6)	<0.01
80–84	551 (72.1)	213 (27.9)	
85–89	297 (69.2)	132 (30.8)	
90+	133 (63.3)	77 (36.7)	
Gender			
Men	689 (73.8)	245 (26.2)	0.16
Women	1027 (71.1)	417 (28.9)	
Education			
>9 years	716 (75.4)	234 (24.6)	<0.01
≤9 years	996 (69.9)	428 (30.1)	
Social class			
Non-manual	812 (74.4)	279 (25.6)	0.01
Manual	887 (69.8)	383 (30.2)	
Rural/urban categories (at the baseline)			
Major Conurbation	384 (70.9)	158 (29.2)	<0.01
Minor Conurbation	332 (66.3)	169 (33.7)	
City and Town	743 (76.8)	224 (23.2)	
Town and Fringe	201 (67.9)	95 (32.1)	
Villages and Dispersed	56 (77.8)	16 (22.2)	
Area deprivation (at the baseline)			
Q1 (least)	453 (75.9)	144 (24.1)	<0.01
Q2	457 (76.6)	140 (23.5)	
Q3	410 (69.4)	181 (30.6)	
Q4 (most)	396 (66.8)	197 (33.2)	

^a^Although this analysis used the age at year-10, the relative difference and the proportion of relocated population should not be affected.

The proportion of relocated population was significantly various across the five rural/urban categories and area deprivation quartiles (Table 2). After adjusting for individual level factors and area deprivation, people living in City and Town areas at the baseline were less likely to relocate their residence in older age [odds ratio (OR): 0.80, 95% confidence interval (CI): 0.61, 1.04] while those living in more rural settings (Town and Fringe) at the baseline were over 30% more likely to move compared with those in Major Conurbations (OR: 1.39, 95% 0.97, 2.00). The likelihood of relocation in older age increased from least to most deprived areas. After controlling for socio-demographic factors, people living in the most deprived areas in 1991 had an over 50% higher risk of moving (Table 2). Both area deprivation and rural/urban categories had independent influence on relocation. A sensitivity analysis using the 2001 version showed that people living in rural areas still had higher likelihood of relocation over 10 years.

**Table 2:** OR of relocation in later life in different socio-demographic and environmental factors

	*Model 1, OR (95% CI)*	**P*-value*	*Model 2, OR (95% CI)*	**P*-value*	*Model 3, OR (95% CI)*	**P*-value*
Age	1.03 (1.01, 1.05)	<0.01			1.03 (1.01, 1.05)	0.01
Gender						
Men (ref.)	1.00 (0.00, 0.00)	0.16			1.00 (0.00, 0.00)	0.15
Women	1.14 (0.95, 1.37)				1.15 (0.95, 1.40)	
Education						
>9 years (ref.)	1.00 (0.00, 0.00)	<0.01			1.00 (0.00, 0.00)	0.46
<9 years	1.31 (1.09, 1.58)				1.09 (0.87, 1.35)	
Social class						
Non-manual (ref.)	1.00 (0.00, 0.00)	0.01			1.00 (0.00, 0.00)	0.36
Manual	1.26 (1.05, 1.51)				1.10 (0.90, 1.36)	
Rural/urban categories (1991)						
Major Conurbation (ref.)	1.00 (0.00, 0.00)	<0.01	1.00 (0.00, 0.00)	<0.01	1.00 (0.00, 0.00)	<0.01
Minor Conurbation	1.23 (0.95, 1.61)		1.20 (0.92, 1.56)		1.21 (0.91, 1.60)	
City and Town	0.73 (0.58, 0.93)		0.76 (0.60, 0.97)		0.80 (0.61, 1.04)	
Town and Fringe	1.15 (0.85, 1.56)		1.15 (0.84, 1.56)		1.39 (0.97, 2.00)	
Villages and Dispersed	0.67 (0.39, 1.25)		0.72 (0.40, 1.29)		0.91 (0.47, 1.73)	
Area deprivation (1991)						
Q1 (least, ref.)	1.00 (0.00, 0.00)	<0.01	1.00 (0.00, 0.00)	<0.01	1.00 (0.00, 0.00)	<0.01
Q2	1.00 (0.74, 1.35)		0.97 (0.72, 1.32)		1.10 (0.81, 1.49)	
Q3	1.48 (1.10, 1.98)		1.41 (1.05, 1.89)		1.55 (1.15, 2.09)	
Q4 (most)	1.68 (1.26, 2.23)		1.55 (1.15, 2.08)		1.66 (1.22, 2.27)	

ref., reference group.

Model 1: Univariate model—the influence of each individual and community level factor.

Model 2: Multivariable model—the influence of community level factors (rural/urban categories or area deprivation) adjusted for individual level factors.

Model 3: Full model—all the individual and community level variables.

### Interaction between urban/rural areas and deprivation score

**Fig. 2:**
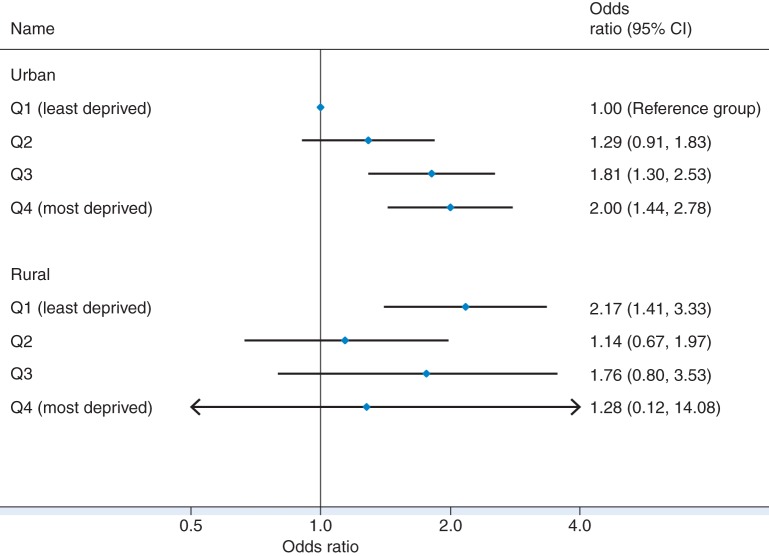
OR of relocation over 10 years from least to most deprived quartiles (baseline) by urban and rural areas (adjusted for age, gender, education and social class).

The association between relocation in later life and area deprivation was found to be different in urban and rural areas. The urban/rural categories significantly modify the association between area deprivation and relocation in later life (Fig. 2). In urban areas, people living in the most deprived areas at the baseline were twice as likely to move in the next 10 years compared with those in the least deprived areas (OR: 2.00, 95% CI: 1.44, 2.78). Older people living in rural areas were more likely to relocate, but the increasing ‘dose–response’ effect from least to most deprived quartiles was less clear compared with those living in urban areas.

## Discussion

### Main finding of this study

Among those who responded to the year-10 interview, nearly one-third had moved their residence after 65 years old and most of them moved within the same rural/urban categories. Several individual level factors including older age, low education and low social class were associated with higher probability of moving and increased from least to most deprived areas particularly in urban settings (OR: 2.00; 95% CI: 1.44, 2.78). Those living in rural areas generally had a higher probability of relocation.

Instead of using self-reported data, this study was able to compare the changes in residential locations at small area level and investigate late-life relocation in large urban and rural areas of England. The results of this study provided more information on relocation in community-based populations.

### What is already known on this topic

Previous UK studies reported the relocation rate in people aged 50 and over was 3.4% during the last 12 months and nearly 30% between censuses.^2,3^ The relationship between relocation and age was U-shaped with higher percentage in early older age (age 50–64) and the oldest old (age 85+).^2^ Qualitative studies have reported that the maintenance of large properties or gardens, access to local services (such as food stores, GP surgery and libraries), quality of infrastructure and safety are considered to be important triggers for relocation in later life.^11,23^

### What this study adds

This study found that community environment had a substantial impact on the probability of relocation at older age. Older people living in rural areas are more likely to change their residential areas over time. Rural communities in England might face substantial challenges meeting the needs of older rural residents. Improvement of service provisions, public transport, home maintenance and adaptations have been highlighted as key issues of policy development for ageing in place in rural areas.^15,16^ Relocation at older age could be related to decline of physical functions but not solely to clinical level illness.^24^ In fact, few older people change their residential address in their last year of life.^25^ Some activities in daily life, such as garden maintenance, grocery shopping and access to healthcare, could be important concerns of relocation at older age. In the literature, moving is sometimes considered to be a pro-active behaviour of coping with ‘environment press’.^5^ A US-based study reports that older people are more likely to live in a distressed neighbourhood but unlikely to move to a secure one compared with younger age groups.^26^ Instead of ‘ageing in place’, some older people might actually be ‘stuck in place’ and suffer from poor quality of community environment. Relocation between urban and rural areas was not a major feature in this study. Although individual choice will be limited by economic status and other factors, older people in England were more likely to move to areas with familiar environmental contexts.

Area deprivation at baseline was particularly important to relocation at older age. The influence of area deprivation on relocation at older age can be discussed from two angles. First, deprivation score could be a proxy for measuring the quality of community environment. More deprived areas are generally related to ‘environmental press’, poor physical (pavement conditions and green space) and social (crime, social capital) features in communities.^27^ Living in these areas could be stressful and substantially restrict daily activities and lead to moving at older age, particularly in urban areas. Although the unclear relationship between deprivation and relocation might be due to small sample size of rural population, the Townsend deprivation score, a compositional measurement of individual/household socioeconomic status, might not reflect the actual quality of local environments in rural areas.

Second, relocation at older age could be related to cumulative influence of individual and community level factors. People living in more deprived areas are more likely to have disadvantaged conditions (difficulties of having stable jobs, property ownership and healthy lifestyle), poor health status.^28–32^ Urban city and town (Cambridgeshire and Oxford), which contained a higher proportion of people with higher socioeconomic status, seemed to provide a more stable environment perhaps with better social supports and networks enabling residents to live in their communities until very old age and thus achieving ageing in place.

The findings of this study might indicate that ‘ageing in place’ is probably closely associated with ‘age-friendly communities’, which focus on enabling active and healthy ageing through improving the age-friendliness of physical (transport, outdoor spaces and buildings) and social (social participation, communication and information, community support and health services) environments in communities.^17,33^ An age-friendly environment may support older people to live actively and independently in their communities and to avoid involuntary relocation. ‘Ageing in place’ and ‘age-friendly environment’ are two relevant policy areas that provide more comprehensive support for older people.

From a research angle, relocation can cause loss to follow-up and substantially affect research findings. In ageing studies, relocation such as moving to institutions is a reasonably frequent event and people living in more deprived areas are more likely to move, making it difficult to trace this population and collect longitudinal data on health status.^34^ Information on relocation might be an important variable to capture in order to improve research quality and clarify the causal direction between place and individual health.

### Limitations of this study

Since detailed addresses and postcodes cannot be released, information on relocation was based on comparison of ED codes in 1991 and 2001. People with ‘the same ED codes’ could have changed their residences within the same EDs, although relocation within geographical units might be less of an issue as such moves can allow location to a more supportive setting without losing social contacts and emotional bonding to a familiar environment.^35^ Multiple relocations could not be identified. Changes in small area level units and mapping techniques might have led to different ED codes or rural/urban categories over 10 years. Owing to limited data on environmental features in the 1990s, this study only used Townsend Deprivation Score, a compositional measurement aggregating household and individual level data, which did not include specific environmental features.

Although this study population included a variety of community environments in England, the findings of this study might have limited generalizability to other countries, given these UK-specific measurements of community environment and special characteristics of the study population. Since the analysis was based on the year-10 follow-up, the problem of attrition, driven by refusal, loss to follow-up as well as death, could have led to an underestimation of relocation, particularly in those living in more deprived areas. This study did not include the measures of health conditions. Although people with poor health conditions at baseline might have a higher risk of relocation, they were also less likely to survive until the year-10 interview.

This study was not able to identify voluntary and involuntary moves and the motivation for relocation. Some people might want to move to residences with special services, care or adaptation for older age but face the dilemma of being away from family and connections in their original communities. Individual characteristics, such as socioeconomic status, health conditions and age, might increase or decrease personal competence and modify the impact of area deprivation on relocation. Reasons for relocation and interactions with individual characteristics need to be explored in future research to provide more comprehensive understanding of relocation at older age.

## Conclusions

This study investigates relocation in older people over a 10-year follow-up and identified two community level factors, urban/rural contexts and area deprivation, which are associated with moving at older age. Community environment could play an important role in supporting ageing in place. Information on relocation at older age including time points, direction and cause of moving needs to be explored in the future research.

## Funding

Medical Research Council Cognitive Function and Ageing Study (MRC CFAS) was funded by the Department of health and the Medical Research Council (grant number G9901400); F.E.M. and A.M.P. are supported by the Medical Research Council (grant number U105292687 and MR/K021907/1); Y.-T.W. received a PhD scholarship from Cambridge Trust, University of Cambridge. We thank the participants, their families, general practitioners and their staff, and the primary care trusts for their cooperation and support.
